# Functional analysis and transcriptional profiling of non-coding RNAs in yeast

**DOI:** 10.1042/BCJ20253069

**Published:** 2025-09-30

**Authors:** Tanda Qi, Daniela Delneri, Soukaina Timouma

**Affiliations:** 1 Manchester Institute of Biotechnology, Faculty of Biology Medicine and Health, The University of Manchester, Manchester, United Kingdom; 2 Division of Evolution, Infection and Genomics, Faculty of Biology, Medicine and Health, The University of Manchester, Manchester, United Kingdom

**Keywords:** *Saccharomyces cerevisiae*, functional genomics, non-coding RNA, transcription, yeast

## Abstract

Advanced transcriptomic technology has identified a great number of non-coding RNAs (ncRNAs) that are pervasively transcribed in the yeast genome. ncRNAs can be classified into short ncRNAs (<200 nt) and long ncRNAs (lncRNAs; >200 nt). Those transcripts are strictly regulated through transcription and degradation mechanisms to maintain proper cellular homeostasis and prevent aberrant expression. It has been revealed that ncRNAs can play roles in various regulatory processes, particularly in transcriptional regulation. While short ncRNAs are well characterised, the function of lncRNAs remains poorly understood. Both functional and transcriptional profiling have been applied to fill the gap in the lncRNA functions landscape. It has been proven by functional profiling that these long transcripts can serve important cellular roles in gene regulation, RNA metabolism, sexual differentiation and telomeric overhang homeostasis. In addition, transcriptional profiling allowed the characterisation of ncRNAs involved in the cell cycle, colony subpopulation dynamics, virulence and regulatory networks. In this review, we introduce the classification, the cellular fate, the evolution and conservation, the mechanisms of action, and the profiling of yeast ncRNAs.

## Introduction

Recent studies have demonstrated that non-coding RNAs (ncRNAs) are not merely transcriptional noise but are involved in various regulatory mechanisms that influence the expression of protein-coding genes. In eukaryotes, several functional ncRNAs have been characterised and were shown to be playing roles in developmental regulation, response to environmental stimuli such as drought and cold stress, dosage compensation, alternative splicing, genomic imprinting, X-chromosome inactivation, transcriptional regulation through interactions with proteins, DNA or other RNAs, among others [1–8].

In yeast, ncRNAs remain a hot topic. As a model organism, *Saccharomyces cerevisiae* possesses a compact, fully-sequenced and well-annotated genome, making it a great host for ncRNA study. It has been revealed that the pervasive transcription of ncRNAs is a well-documented phenomenon in yeast. Up to 85% of the *S. cerevisiae* genome is transcribed [9,10]. Among these transcripts, approximately 30% are non-coding, which is notably lower than in humans (98.5%), plants (>60%), or metazoans (>75%) but higher than in prokaryotes (less than 25%) [11–14]. A recent study revealed that 53% of *S. cerevisiae* RNAPII-associated nascent transcripts are non-coding [15]. Among these ncRNAs, short ncRNAs [i.e. rRNAs, tRNAs, small nuclear RNAs (snRNAs) and small nucleolar RNAs (snoRNAs)] are well-characterised housekeeping transcripts, while lncRNAs have less understood mechanisms of action (see section on ‘Classes of ncRNAs in yeast’). The ncRNA transcription can be supported by the three RNA polymerases (i.e. I, II and III) according to their classes [16]. The RNA polymerase I transcribes most of the rRNAs except for the 5S rRNA, while the RNA polymerase II transcribes primarily the snRNAs, snoRNAs, mRNA and ncRNA of pervasive transcription. RNA polymerase III transcribes the tRNAs, 5S rRNA and other small RNAs. This transcriptional activity is not random; rather, it is regulated by specific pathways, such as the Nrd1-Nab3-Sen1 (NNS) complex or cleavage and polyadenylation/cleavage factor (CPF-CF) complex, which is crucial for the termination of ncRNA transcription [17,18]. Such termination pathway ensures that ncRNAs are properly processed and degraded, preventing the accumulation of potentially harmful transcripts that could disrupt cellular functions [19].

The functional and transcriptional profiling of lncRNAs in yeast has emerged as a critical area of research that has the potential to reveal the complex roles these molecules play in gene regulation and cellular processes. One of the primary functions of lncRNAs in yeast is their involvement in transcriptional regulation, via transcriptional interference (TI) and chromatin remodelling, for example [20,21], highlighting the importance of lncRNAs in maintaining the integrity of gene expression networks. Single-cell RNA-seq has revealed that lncRNA expression can vary between individual cells, even within the same population [22], suggesting that lncRNAs may have more complicated specialised functions in distinct cellular contexts. In addition to transcriptional regulation, lncRNAs also play important roles in specific developmental processes in budding yeast, such as meiosis [23,24]. This regulatory capacity suggests that lncRNAs are essential for the proper execution of developmental programmes in yeast. Given that some lncRNAs exhibit conservation across species (for example, TI by lncRNAs and chromatin remodelling driven by lncRNA activity are found in both yeast and mammals; see section on ‘Evolution and conservation of lncRNA’), their characterisation in yeast can broaden our understanding of ncRNA-related regulatory mechanisms across higher eukaryotes.

In this review, we introduce all the existing ncRNA classes and subclasses in yeast, as well as their discovery and classification. With an emphasis on lncRNAs that are less well characterised, we discuss their transcription and degradation mechanisms, as well as their evolutionary conservation and mechanisms of action in *cis-* and *trans-*regulatory functions. We also present recent studies on functional and transcriptional profiling, revealing the involvement of ncRNAs in a variety of cellular processes and gene regulation. Furthermore, we highlight groundbreaking findings that challenge the ‘non-coding’ label, demonstrating that some ncRNAs are also translated into peptides.

## Classes of ncRNAs in yeast

Yeast ncRNAs can be divided into two major classes, which include short ncRNAs that have a length below 200 nucleotides, and lncRNAs that are composed of more than 200 nucleotides.

**Figure 1:**
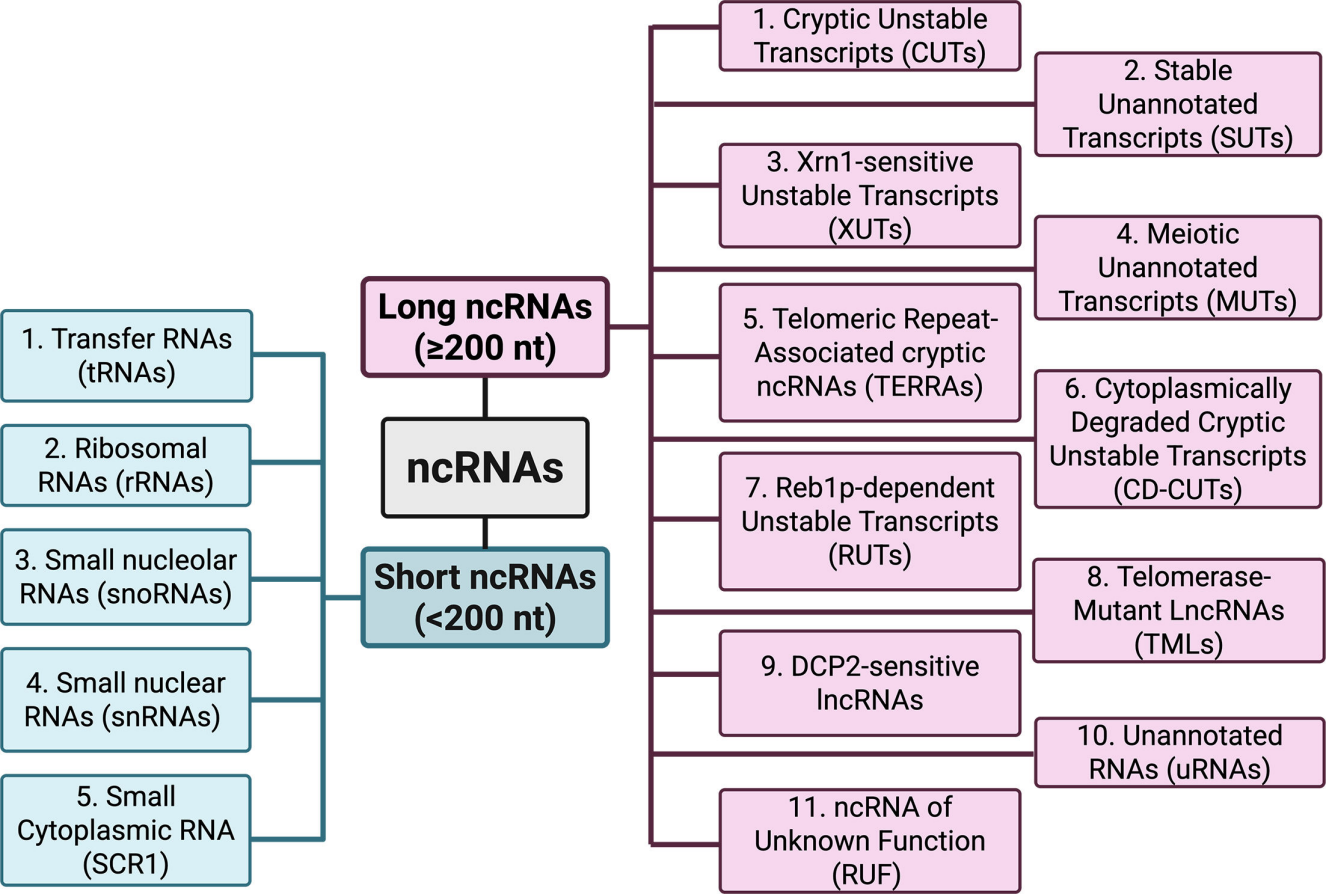
Classification of ncRNAs in *S. cerevisiae.* ncRNAs can be divided into short and long ncRNAs. There are 5 classes of short ncRNAs and 11 classes of long ncRNAs in *S. cerevisiae*. The figure was created using BioRender (https://biorender.com/)*.*

In *S. cerevisiae*, short ncRNAs include tRNAs, rRNAs, snoRNAs, snRNAs and the small cytoplasmic RNA (*SCR1*) (Figure 1). For instance, tRNAs and rRNAs are two well-known ncRNA sub-classes that are responsible for protein biosynthesis. Specifically, tRNAs serve as adaptors that translate the codon from mRNA into amino acids (aa) during protein synthesis [25], while rRNAs dock to ribosomal proteins and form subunits of protein-synthesising organelles (i.e. ribosomes), guiding and catalysing peptide bond formation [26]. snoRNAs are localised in the nucleolus and play a role in the processing and modification of a variety of RNAs, including rRNAs, snRNAs and mRNAs [27–29]. According to the conserved regions present in the sequence, yeast snoRNAs are categorised into C/D box snoRNAs [30] and H/ACA box snoRNAs [31]. Each group of snoRNAs can be assembled with their associated proteins to form stable ribonucleoprotein for further RNA processing [32]. snRNAs are another sub-class of short ncRNAs that are located in the nucleus [33]. Similarly to snoRNAs, snRNAs also assemble with proteins to form small nuclear ribonucleoproteins, which act as subunits of the spliceosome [34]. snRNAs participate in the pre-mRNA splicing, specifically via the recognition of 5′ splice site on the pre-mRNA [35]. *SCR1* is the RNA subunit of the signal recognition particle, an evolutionarily conserved ribonucleoprotein that plays a role in co-translational protein targeting to the endoplasmic reticulum [36,37]. Notably, some short ncRNAs discovered in other eukaryotes, such as miRNAs and siRNAs, are absent from *S. cerevisiae* due to the loss of RNAi pathway [38]. However, alternative gene repression mechanisms exist in *S. cerevisiae*, such as the dose-dependent co-repression mediated by antisense lncRNAs [39,40]. RNAi mechanisms exist in other budding yeasts, such as *Naumovozyma castellii* and *Candida albicans* [38], and in the fission yeast *Schizosaccharomyces pombe* [41].

Compared with short ncRNAs, lncRNAs are ‘non-conventional’ ncRNAs for which the mechanisms of action are less well understood. To date, 11 subclasses of lncRNAs have been discovered in *S. cerevisiae* (Figure 1). Specifically, there are (i) cryptic unstable transcripts (CUTs), transcripts with a short half-life that are rapidly degraded by the nuclear exosome [42,43]; (ii) stable unannotated transcripts (SUTs), transcripts that are stable and transcribed from unannotated regions [42]; (iii) Xrn1-sensitive unstable transcripts (XUTs), transcripts that are sensitive to degradation by the Xrn1p exonuclease [44]; (iv) meiotic unannotated transcripts (MUTs), transcripts expressed during meiosis and associated with the regulation of meiotic processes [24]; (v) telomeric repeat associated cryptic ncRNAs (TERRAs), transcripts transcribed from telomeric repeat regions that are associated with telomere maintenance [45]; (vi) cytoplasmically degraded cryptic unstable transcripts (CD-CUTs), transcripts that are sensitive to the cytoplasmic nonsense-mediated decay (NMD) pathway [46]; (vii) Reb1p-dependent unstable transcripts (RUTs), transcripts derived from Reb1p-dependent termination events [47]; (viii) telomerase-mutant lncRNAs (TMLs), transcripts that are up-regulated in telomerase-negative cells [48]; (viiii) DCP2-sensitive lncRNAs, novel transcripts that are degraded by the decapping protein DCP2 through a pathway that is independent of mRNA decapping regulators [49]; (x) unannotated RNAs (uRNAs), transcripts that were previously unclassified and reported in 2014 [50]; and finally (xi) transcripts of ncRNA of unknown function (RUF) [51]. The boundaries between different classes of lncRNAs are often unclear, resulting in great overlaps between classes [44,52,53]. For example, a portion of CUTs (20%) and SUTs (75%) has been redefined as XUTs [44]. Since uRNAs and XUTs are sensitive to NMD pathway [44,50], they can be described as CD-CUTs. Besides, lncRNA isoforms can be recognised by different RNA surveillance pathways, which impacts on their classification [54]. Moreover, lncRNAs can be further grouped by their location in the genome as antisense, intronic, intergenic or enhancer transcripts [42–44,55].

In addition to *S. cerevisiae*, lncRNAs have also been recently identified in other budding and fission yeast species. Specifically, CUTs and XUTs have been found in *N. castellii* and in the fission yeast *S. pombe* [56–60]. *N. castellii* and *S. pombe* also possess Dicer-sensitive unstable transcripts (DUTs) which are absent from *S. cerevisiae,* which lacks the ribonuclease III Dicer and the RNAi machinery [56,57].

## Discovery of novel ncRNAs

The history of ncRNA discovery is intricately tied to the evolution of technological advancements in molecular biology over the past decades. There have been several methods employed that allowed ncRNA discovery and classification.

First, once whole genome sequences became available, novel ncRNAs can be identified through homologous sequence prediction based on sequence alignment of known ncRNAs and conserved motif features. For example, this method allowed the discovery of members of the 2′-O-methylation guide snoRNA class [61]. Recently, deep learning technology has been used to predict ncRNAs in the human genome and to assist their classifications using primary sequence information [62,63]. Such approaches allow the discovery of new ncRNAs.

Moreover, the advent of high-throughput RNA-seq and methods for data analysis has revolutionised our understanding of the transcriptome, enabling researchers to detect previously unannotated transcripts across various organisms, including yeast. This technology has allowed the identification of a substantial portion of ncRNAs in the yeast genome, challenging traditional views that primarily considered these regions as non-functional ‘junk’ DNA [64]. For example, the comparison of transcripts extracted from a telomerase-negative yeast strain and a wildtype strain highlighted 112 uncharacterised transcripts (~467 nucleotides in length), fired from non-intergenic regions, which showed differential expression. This observation led to the identification of a new subclass of lncRNA, referred to as TMLs [48].

The discovery of ncRNAs has also been possible with the use of gene knockout techniques. Specifically, by inactivating the RNA degradation pathways, ncRNA transcripts remain in the cell longer and can be detected. For example, since CUTs and MUTs are rapidly degraded by RNA turnover mechanisms, they were discovered thanks to the knockout of one of the genes involved in their degradation (nuclear exosome), namely the *RRP6* [24,43]. Additionally, TERRA ncRNAs were discovered by knocking out *RAT1* (encoding for nuclear 5′ to 3′ RNA exoribonuclease) [45], while CD-CUTs ncRNAs were identified in nonsense-mediated mRNA decay (NMD) mutants [46]. XUTs were discovered by repressing *XNR1* encoding for a cytoplasmic 5′–3′ exonuclease [44]; DCP2-sensitive lncRNAs were discovered in a decapping enzyme deficient strain (dcp2Δ) [49]; and RUTs were revealed following the discovery of an additional termination pathway in yeast mediated by *REB1* (road-block termination) [47]. In summary, identifying and uncovering RNA processing-related pathways has proven to be crucial to discovering novel classes of ncRNAs.

## Initiation and termination of lncRNA transcription in the cell

Effective and accurate synthesis of ncRNAs is critical, given their essential regulatory roles to ensure cellular function and stability. Precise transcription not only ensures the correct production of functional ncRNA molecules but also enables transcription-mediated mechanisms, such as transcriptional insulation and interference, that contribute to the activation, repression or switching of gene expression, particularly within gene clusters [65–70]. For proper gene expression, steps such as initiation and termination of transcription must be precise and strictly supervised.

**Figure 2:**
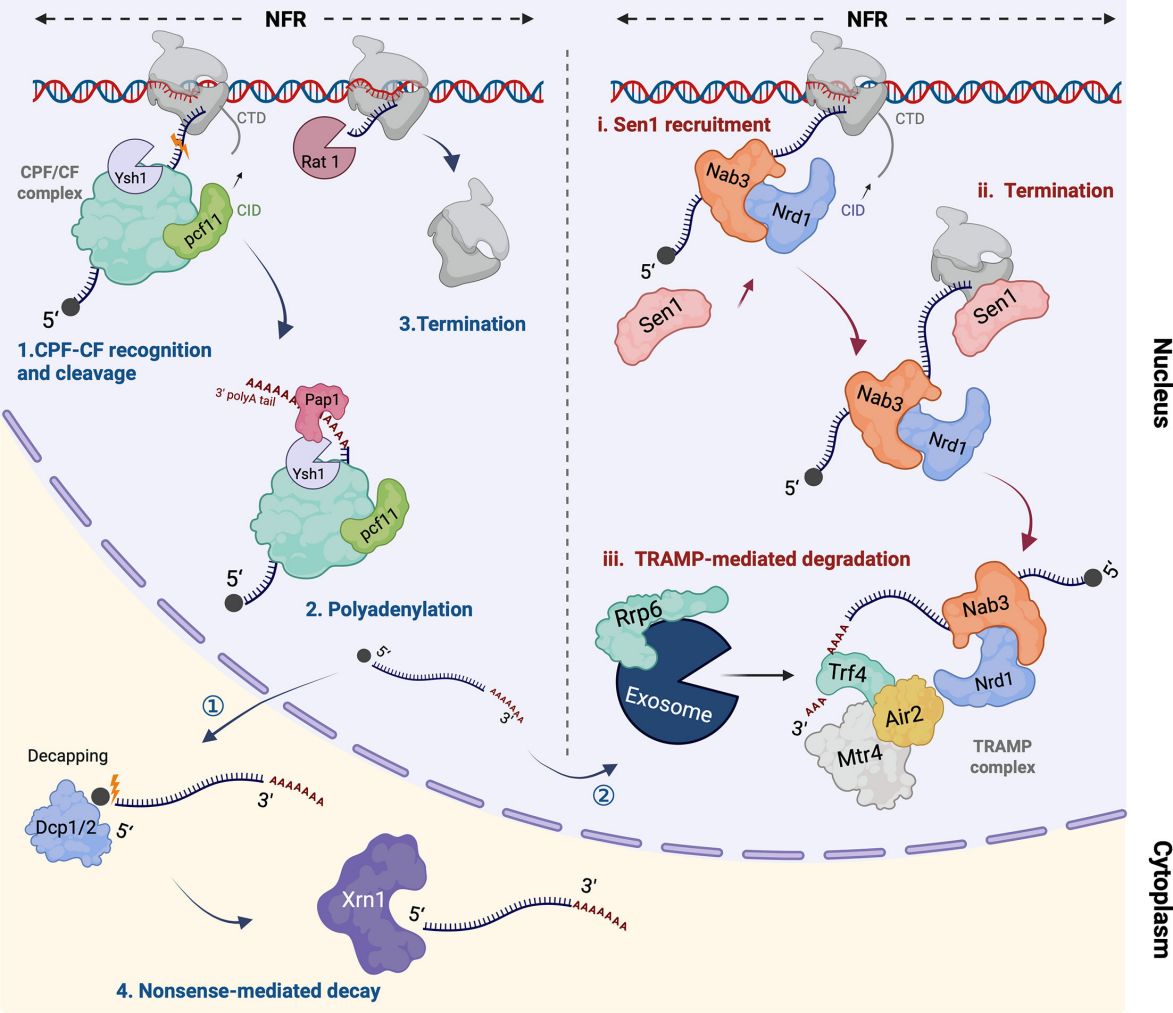
Cellular fate of lncRNAs in yeast. lncRNAs are transcribed from the NFR within the genome. Their transcriptional termination occurs via two main pathways: the CPF-CF pathway (blue colour, numbered with 1, 2 and 3) or the NNS pathway (red coloured, numbered with i and ii). Following termination, these lncRNAs are typically targeted for degradation through either the NMD pathway (blue coloured, numbered with 4) or TRAMP complex-mediated degradation (red coloured, numbered with iii) respectively. Some lncRNAs terminated by the CPF-CF pathway are also degraded by nucleus exosome. The figure was created using BioRender (https://biorender.com/)*.*

Pervasive lncRNAs in *S. cerevisiae* mostly originate from nucleosome-free areas (NFRs), also called nucleosome-depleted regions (Figure 2) [42,43]. There are four classes of NFRs in the yeast genome, namely 5′ NFR [71], 3′ NFR [42], open reading frame NFRs (ORF-NFRs) and other-NFRs [72].

Two-thirds of unannotated ncRNA transcription is transcribed from the 5′ NFR around the transcription start site (TSS) [21] . Specifically, they can be transcribed with the bidirectional promoter shared with other genes, creating either sense-orientated transcripts that overlap with the sense gene, or antisense-orientated transcripts without overlapping the sense genes [42]. However, some antisense lncRNAs can originate from the 3′ NFRs around the transcription termination site in the tandem intergenic region, which encompasses the 3′ end of one gene and the 5′ end of another same-direction gene. These lncRNAs are regulated by a 5′ promoter of the adjacent gene or by a hidden antisense promoter [73]. To date, there is no evidence that there are lncRNAs transcribed from ORF-NFR and other-NFR regions since their discovery [72].

The repression of lncRNA transcription associated with NFRs can be regulated through an *ISW2*-mediated repression. *ISW2* is a catalytic component of the ISW2 protein complex, which plays a role in the remodelling of the chromatin by catalysing an ATP-dependent alteration in the structure of nucleosome DNA. By sliding the nucleosomes towards the NFRs and consequently changing the size of the NFR, the ISW2 protein complex limits the access of the transcription factors, including polymerases, to the TSS of lncRNAs, thus repressing the lncRNAs transcription [72].

Beyond transcription initiation, termination of transcription is a crucial interconnected process that ensures accurate gene expression in yeast. While transcription initiation establishes the start of RNA synthesis, effective termination mechanisms are essential for releasing RNA polymerase from the DNA template at the correct point in time, setting the stage for subsequent transcription cycles and element recycling [19].

Two independent pathways are employed for lncRNA transcription termination (Figure 2). On the one hand, the CPF-CF complex terminates the transcription by recognising the AU-rich polyadenylation signal within the nascent lncRNA precursor. The complex also binds to RNA polymerase II via its subunit Pcf11p, which contains an N-terminal CTD interaction domain (CID) that recognises the C-terminal domain (CTD) of RNA polymerase II subunit. Once bound to the RNA, the Ysh1p subunit of the CPF-CF complex, which possesses an endonuclease activity, cleaves the RNA transcript at the polyadenylation site. The cleaved RNA is further processed by the poly(A) polymerase Pap1p, which enables the addition of a poly(A) tail to the 3′ end of the nascent RNA [74]. After transcript cleavage, RNA polymerase II continues transcription but is eventually released from the DNA template through a not fully understood mechanism. One model suggested that after transcript cleavage, the remaining 3′ fragment attached to RNAPII is degraded by the 5′ to 3′ exonuclease Rat1, which subsequently facilitates the disassembly of the elongation complex [75]. CPF-CF complex termination of transcription is used by budding yeast for the SUTs and XUTs transcripts [55].

On the other hand, other lncRNAs, such as CUTs and NUTs, are terminated by the NNS complex [55,76]. Unlike the CPF-CF pathway, which involves RNA cleavage before RNA polymerase II detaching from DNA, the NNS degradation pathway does not cleave RNA during the whole termination process. The NNS complex terminates the transcription starting with binding the proteins Nrd1p and Nab3p to short motifs (GUAA/ G for Nrd1p and UCUUG for Nab3p) that are present in the nascent RNA. Similarly to the CPF-CF pathway, the NNS complex can interact directly with the RNA polymerase II, particularly through the recognition of the RNA polymerase II CTD by the CID of Nrd1p. Next, the Sen1 protein, an ATP-dependent helicase, is recruited to terminate the transcription by dissociating the RNA polymerase II from the elongation complex [77]. Notably, the termination by the NNS complex forms a non-poly(A) 3′ end in the nascent RNA, which assists in further degradation steps, including polyadenylation modification by TRAMP (Trf4/Air2/Mtr4 polyadenylation complex) and recruitment of the exosome [78–80]. The transcriptional termination by the NNS complex is very efficient [80]. As a result, non-coding transcripts associated with this pathway rarely exhibit transcriptional read-through, in contrast with SUTs, which are terminated by the CPF-CF complex and are more prone to read-through events [54]. Such an efficient termination mechanism is employed to insulate downstream genes from upstream TI (for example, it is the case for CUT60 [65]. The NNS complex has also been reported to play a key role in the regulatory network between antisense lncRNAs and their corresponding sense genes by promoting early transcription termination of antisense lncRNAs [81]. Such early termination limits their elongation and reduces antisense transcription rate, thereby countering the repression on the sense gene [82].

Interestingly, the lncRNAs terminated by the CPF-CF complex are usually rapidly exported from the nucleus to the cytoplasm, while NNS transcription termination is limited to the nucleus RNA degradation pathway with the exosome/Rrp6p and TRAMP, resulting in a shorter half-life for these lncRNAs. This distinction in lncRNA product stability may provide insights into the potential functional dynamic of these two classes of lncRNAs terminated by two different mechanisms.

## lncRNA degradation pathway

Yeasts exhibit pervasive transcription that leads to ncRNA spurious transcription or ncRNA by-product transcription [55]. Thus, there are several robust degradation machineries employed by the cell to control its ncRNA levels. So far, two ncRNA degradation machineries have been identified in yeast for ncRNAs, namely the 5′-end Xrn1-dependent cytoplasmic RNA degradation and the 3′-end exosome-dependent nuclear RNA degradation (Figure 2) [55].

The cytoplasmic RNA degradation is a general machinery for RNA turnover, targeting especially mature mRNAs but also lncRNAs such as SUTs, XUTs and some CUTs (*SRG1*, *Ty1 RTL*, among others) [55]. These lncRNAs exhibit mRNA-like features, such as 3′ cleavage and polyadenylation, allowing them to escape nuclear RNA surveillance and be exported into the cytoplasm [83]. The cytoplasmic degradation of a large proportion of these lncRNAs is mediated by the NMD pathway (Figure 2). In this pathway, the lncRNA degradation starts with RNA decapping by Dcp1/2 decapping enzyme complex [84], followed by 5′-3′ degradation by Xrn1p. Notably, it is reported that a subset of antisense XUTs, namely NMD-insensitive XUTs, can escape from NMD by forming double-stranded RNA with their corresponding sense RNA [53]. Further degradation is suggested to occur with the assistance of the RNA helicases Mtr4 and Dbp2 [85], which facilitate the destabilisation of antisense XUTs, likely through double-stranded RNA unwinding [53], or via Xrn1p-dependent co-translational RNA degradation [86]. The NMD pathway is crucial in facilitating the degradation of aberrant transcripts, thereby preventing the buildup of truncated polypeptides that are typically non-functional and may exert harmful effects on cellular processes [50,87].

In contrast, the nucleus RNA degradation occurs when the NNS transcription termination is employed. Several unstable nucleus transcripts are degraded in the nucleus with this mechanism, especially CUTs [80]. After NNS-mediated termination of transcription, the transcripts are polyadenylated on the 3′ end by Trf4p (β-like nucleotidyltransferases) of the TRAMP complex (Figure 2) [88]. The newly formed short poly(A) tail will serve as an attachment site for the exosome [78]. It has been shown that a knockout of Trf4 leads to a lack of 3′ polyadenylation, resulting in the accumulation of stable transcripts [88], highlighting the role of the poly(A) tails in nucleus ncRNA degradation. For that reason, this process is also called polyadenylation-assisted RNA decay machinery. The short-adenylated ncRNAs are targeted by the nuclear exosome, facilitated by Rrp6p, for 3′ to 5′ degradation [89]. The Mtr4p helicase, associated with the TRAMP complex, facilitates the entry of ncRNA transcripts into the exosome, ensuring efficient RNA processing and degradation [90]. Remarkably, some SUTs are also degraded in the nucleus by exosome, though the majority is degraded in cytoplasm [55].

Timely degradation of ncRNAs is important for the RNA homeostasis *in vivo*. Excessive ncRNA can result in negative effects on the cell by occupying regulatory elements, disturbing transcriptional regulation or affecting chromatin structure. For example, ncRNA degradation by the exosome is required to free the NNS complex subunits, Nrd1 and Nab3, which are recruited on the transcript after termination to participate in subsequent rounds of RNA surveillance and processing [19]. Additionally, ncRNAs, such as promoter-associated RNA, can interfere with transcription by forming RNA/DNA hybrids (R-loops) [91] or potentially occupy transcription factors, as has been observed in humans [92].

## Evolution and conservation of lncRNAs

Most lncRNAs show low primary sequence conservation in eukaryotes [93], including yeast such as *Saccharomyces* sp. [94] and *Candida* sp. [95]*.* The lack of conservation in the primary sequence of lncRNAs reflects the rapid evolution of these transcripts which could suggest their role in specific cellular contexts or environmental conditions. However, there are some examples of conserved lncRNAs across different species, such as metastasis-associated lung adenocarcinoma transcript 1, which is highly conserved in mammals [96], or Tcl1 upstream neuron-associated long intergenic ncRNA (lincRNA) in vertebrates (TUNA) [97], or TERRA in eukaryotes [98].

In contrast with the low conservation in the primary sequence, secondary structures of lncRNAs exhibit a more conserved pattern across closely related species [99]. It has been observed that functional module elements such as hairpin-like structures and stem-loops present poorly conserved primary sequences, while their secondary structure is more conserved [100]. For example, the steroid receptor RNA activator lncRNAs in mammals exhibit an uneven conservation pattern, with the bases of the lncRNAs in the terminal loops, in the bulges and in the looping regions highly conserved (i.e. hence ensuring a conserved secondary structure), while the remaining lncRNA sequences appear divergent [100]. The interspecies conservation of lncRNA secondary structures has been used to predict or screen for functional lncRNAs over yeast species, helping the discovery of functional lncRNA in less-annotated species [101].

There is evidence that some lncRNAs with low primary sequence conservation can still be conserved in terms of expression patterns and syntenic arrangement. This is the case of a study on six polyadenylated antisense transcripts from five yeast species (*S. paradoxus*, *S. mikatae*, *S. bayanus*, *N. castellii* and *Kluyveromyces lactis*) [102]. It was revealed that a conserved expression pattern, such as anti-correlation in expression levels of the sense and antisense transcripts, was observed in at least one combination of these yeast species pairs. A similar study on polyadenylated transcripts in fission yeasts has shown that 129 out of 338 intergenic lncRNAs are conserved in genomic positioning across two or more other genomes despite their low conservation [103]. Some lncRNAs in the *Candida* clade exhibit syntenic conservation and share sequence motifs, though possessing low sequence conservation [95].

Across the yeast clade, including the budding yeasts *S. cerevisiae* and *N. castellii* as well as fission yeast *S. pombe,* the conserved lncRNAs in terms of class include nuclear exosome-sensitive lncRNAs and cytoplasmic Xrn1p-sensitive lncRNAs, corresponding to CUTs and XUTs in *S. cerevisiae* [56,59,60]. Most of the CUTs and XUTs are antisense to the corresponding protein-coding sense genes in this species, i.e. antisense lncRNAs. Remarkably, the antisense lncRNA expression levels in species belonging to the *Saccharomyces* genus (especially *S. cerevisiae*) are higher compared with *N. castellii* [39]. Such a global increase in antisense lncRNA levels is proposed to be caused by the loss of RNAi in *S. cerevisiae*, specifically by the loss of ribonuclease III Dicer [39]. It was reported that antisense lncRNA levels in the *Saccharomyces* genus were found to be correlated with the phylogenetic proximity to *S. cerevisiae,* which possesses the highest levels of antisense lncRNAs. The presence of RNAi in a cell with high levels of antisense lncRNAs has been shown to be disadvantageous in *S. cerevisiae* [39] because double-stranded DNAs, which are formed by hybridisation between mRNA and antisense lncRNAs, are processed by the RNAi machinery, which disrupts both mRNA and antisense lncRNA transcripts. Therefore, the increase in antisense lncRNAs in *Saccharomyces* lineage could be an event related to the loss of the RNAi mechanism. Compared with *S. cerevisiae*, antisense lncRNAs in *N. castellii* primarily consist of a larger proportion of exosome-sensitive antisense transcripts (i.e. CUTs), and a smaller proportion of cytoplasmic lncRNAs that are sensitive to Xrn1p (i.e. XUTs) [57]. The CUTs are located in the nucleus and are rapidly degraded via nuclear RNA surveillance mechanisms. Thus, they are not forming cytoplasmic double-stranded RNAs to be subjected to cytoplasmic Dicer degradation. On the other hand, the XUTs are able to form double-stranded RNA with mRNA and consequently be processed by Dicer if they have escaped from Xrn1p degradation. As a result, it is suggested that *N. castellii* possesses more stringent nuclear RNA surveillance machinery in order to restrict the level of antisense lncRNAs in the cytoplasm. In contrast, the absence of RNAi in *S. cerevisiae* may have facilitated the expansion of the Xrn1-sensitive antisense transcriptome, which has reduced the necessity to retain antisense lncRNAs within the nucleus and consequently led to a decline in CUTs abundance. It remains an open question whether CUTs and XUTs share an evolutionary origin, with their differing abundances across species potentially resulting from variations in the stringency of RNA surveillance mechanisms.

Remarkably, antisense transcripts can serve as a source of *de novo* gene birth, illustrating how new protein-coding genes may emerge from previously non-coding regions. For example, the *MDF1* gene, specific to *S. cerevisiae,* is located on the opposite strand of *ADF1*. In other yeast clades, the locus of *MDF1* is non-coding. This suggests that *MDF1* functioned as an ncRNA before acquiring coding capability—a process highlighting the potential of antisense transcripts to evolve new coding functions [104]. This example underscores the evolutionary flexibility of antisense transcription and its possible role in expanding genomic novelty and organismal adaptability.

## Mechanisms of lncRNA regulatory function in *S. cerevisiae* in *cis*

It is now clear that lncRNAs play important roles in various regulatory processes, particularly in transcriptional regulation, which can primarily be categorised into two mechanisms of action: *cis-*regulation and *trans*-regulation [94,105]. *Cis*-acting lncRNAs are located nearby the gene they regulate and carry the regulatory function by influencing the transcription of the sense gene on the same locus or of neighbouring genes [106]. The majority of lncRNAs regulate genes in *cis*. Examples of *cis-*regulation include mechanisms such as TI, regulatory complexes recruitment and chromatin structure changes.

**Figure 3:**
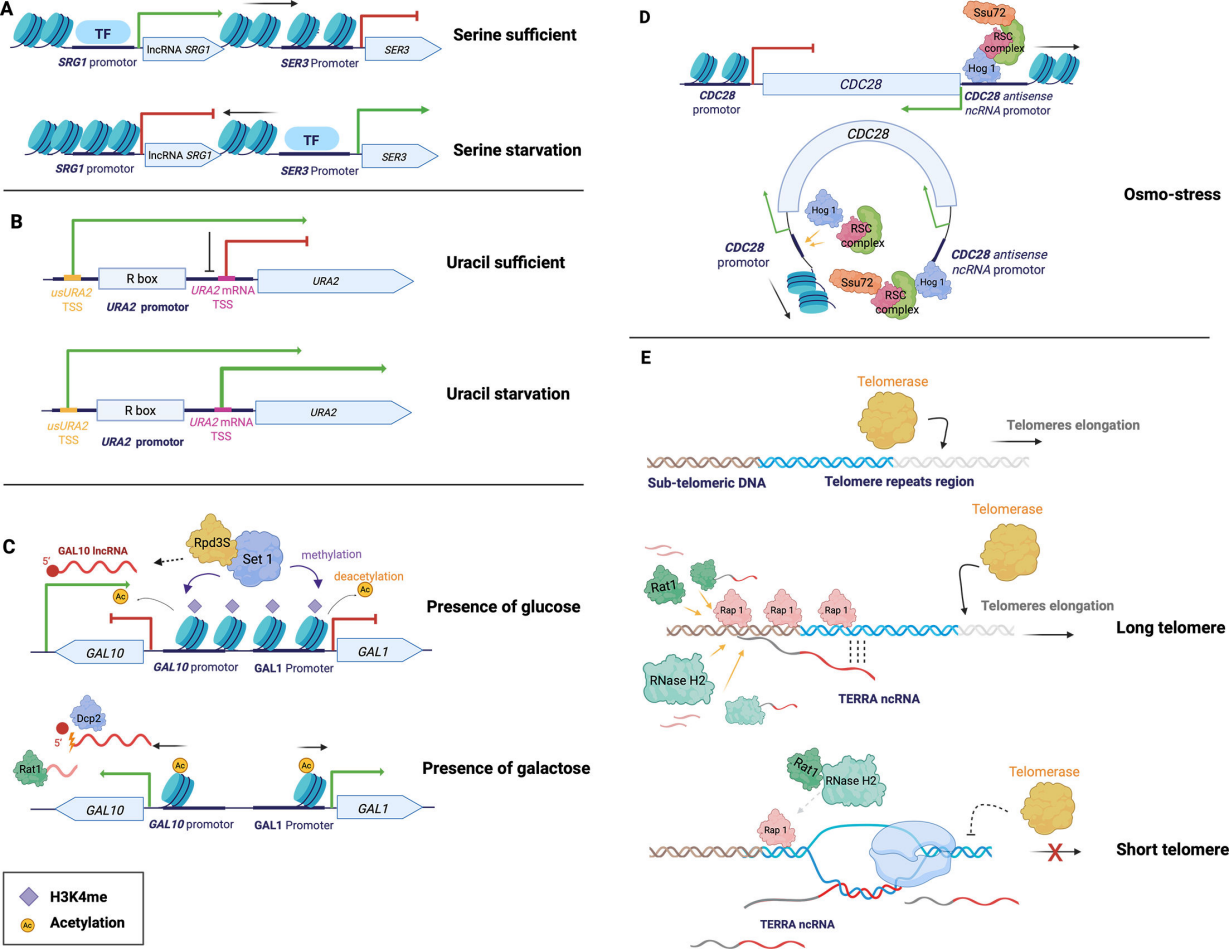
Mechanisms of *cis*-regulatory lncRNAs involved in *S. cerevisiae*. (**A**) Transcriptional interference via nucleosome displacement. In an environment containing the amino acid serine, the expression of the lncRNA *SRG1* is induced and inhibits the expression of *SER3* by rearranging the nucleosomes onto the *SER3* promoter. In contrast, when the cell is starved from serine, the transcription of *SRG1* is switched off, releasing the *SER3* promoter and resulting in the transcription activation of *SER3*. (**B**) Transcriptional interference via alternative transcription initiation. Both transcripts originate from the same promoter but use distinct TSSs: *usURA2* initiates from a weak upstream TSS, while *URA2* mRNA uses a stronger downstream TSS. Under uracil-rich conditions, the mRNA-specific TSS is repressed, favouring *usURA2* transcription and thereby repressing *URA2* expression. During uracil starvation, the downstream TSS is activated, overcoming interference from *usURA2* and promoting *URA2* mRNA transcription. (**C**) Gene repression via recruiting regulatory elements. *GAL10* lncRNA recruits methyltransferase Set1p and Rpd3S deacetylase complex that induces histone modifications on the GAL locus, repressing *GAL1* and *GAL10*. The expression of the *GAL10* and *GAL1* genes is restored after *GAL10* lncRNA is decapped by Dcp2p and degraded by Rat1p. (**D**) Gene activation via gene looping. During osmo-stress, the transcription of *CDC28* antisense lncRNA recruits the transcription activator Hog1p and RSC complex, as well as the gene-looping factor Ssu72p onto its promoter region (i.e. the 3′UTR of *CDC28*). Ssu72p mediates the gene looping between the +1 nucleosome region and the 3′UTR of *CDC28*, facilitating the relocating of Hog1p and RSC complex on the +1 nucleosome region of *CDC28,* resulting in *CDC28* transcription*.* (**E**) Inhibition of the access of genomic elements via R-loop formation. TERRA is initiated from the sub-telomeric regions that contain a complementary sequence with the telomere repeated elements, which allows the formation of an R-loop with the telomere. On the one hand, TERRA is rapidly degraded when a telomere is long because of the presence of abundant Rap1p*,* which recruits the nucleases Rat1p and RNase H2. On the other hand, when a telomere is short, there are fewer Rap1p docked to the telomere, thus resulting in fewer nucleases recruited and an increased level of TERRA. Sufficient TERRA in the cell promotes the formation of R-loop with the telomere and prevents the access of telomerase, consequently inhibiting the telomere elongation. The figure was created using BioRender (https://biorender.com/).

Many lncRNAs are byproducts of TI, in which case these lncRNA transcripts may be either functional or non-functional since this mechanism of expression has not yet been studied at the evolutionary scale in eukaryotes. For example, the lncRNA *SRG1* represses *SER3,* which encodes the serine biosynthetic enzyme 3-phosphoglycerate dehydrogenase (Figure 3, Panel A) [68,69]. The transcription of *SRG1* is activated in an environment containing the amino acid serine. The arrangement of nucleosomes is remodelled to occupy the *SER3* promoter, blocking the interaction between *SER3* and its transcription factor and thus repressing the expression of *SER3* [107]*.* In contrast, when the cell is starved from serine, transcription of *SRG1* is switched off, releasing the *SER3* promoter and thus resulting in the transcription activation of *SER3*. It has been shown that the integration of the *SRG1* promoter into the *GAL7* gene, a gene involved in galactose catabolism, still results in significant suppression of *GAL7* expression when serine is sufficient [69], suggesting that it is the transcription of *SRG1*, rather than the *SRG1* transcript itself, that is responsible for the *SRG1*-mediated repression of *SER3* [69]*.*

Another case of TI is alternative transcription initiation. For example, it was indeed observed between *URA2* (encoding for the carbamoylphosphate synthetase-aspartate transcarbamylase complex) and its CUT lncRNA *usURA2* that is located in the 5′ region of *URA2* [70]*.* Both the *usURA2* and *URA2* mRNA originate from the same promoter but have distinct TSS, with *usURA2* initiating from an upstream weaker TSS and *URA2* from a downstream stronger TSS (Figure 3, Panel B). Under uracil-replete conditions, downstream mRNA-specific TSS is inactivated. As a result, transcription primarily initiates at upstream TSS, leading to *usURA2* transcription and the repression of *URA2* mRNA transcription. In contrast, during uracil starvation, strong downstream mRNA-specific TSS is activated for RNAPII recruitment, counteracting the repression by *usURA2* transcription*,* thus promoting *URA2* mRNA transcription. A similar nucleotide concentration-dependent initiation case was also reported for *IMD2* and its associated CUT (also called attenuated transcripts) [108]. Specifically, the transcription of CUTs or *IMD2* mRNA was regulated based on GTP availability, occurring under GTP-depleted or GTP-replete conditions, respectively. The proposed mechanisms include competition for available RNAPII [108] and RNAPII’s preference for different TSS depending on nucleotide concentrations [70].

In addition, there is evidence that some lncRNAs recruit regulatory elements, particularly chromatin remodelling factors, to perform their *cis*-regulation mechanism, which has been widely observed among antisense lncRNAs [65,109–112]. For example, this is the case for *GAL10* antisense lncRNA (also named SUT013), which regulates yeast growth response to galactose [110]*. GAL10* lncRNA is a Dcp2-sensitive transcript that originates from the 3′ UTR of *GAL10* and overlaps the *GAL10* and *GAL1* ORFs (Figure 3, Panel B). In the presence of glucose, *GAL10* lncRNA is transcribed and induces H3K36me3 and histone deacetylation by recruiting the methyltransferase Set1p and Rpd3S histone deacetylase complex on the entire GAL locus, forming a tight chromatin structure which results in the silencing of *GAL1* and *GAL10* [110]*.* When galactose is present, *GAL10* lncRNA undergoes decapping by the Dcp2 enzyme and is subsequently degraded by the nuclear 5′ → 3′ exonuclease Rat1, which restores the expression of the *GAL10* and *GAL1* genes. Such *cis*-regulation on *GAL* loci by *GAL10* lncRNA transcription is vital to prevent transcriptional leakage of *GAL1* and *GAL10*, which will result in unwanted metabolic switching and fitness defect [113]. Similarly, the lncRNA IME1 regulatory transcript 1 (*IRT1*) represses the expression of the meiosis regulator gene *IME1* and *IME4* by recruiting Set2p (histone methyltransferase) and Set3p (histone deacetylase) [112].

When lncRNA transcripts are required for gene looping, another mechanism of lncRNA *cis*-regulation of genes in yeast is observed (Figure 3, Panel C) [114]. Unlike in animals, where chromatin looping is mediated by CTCF, a protein that interacts with R-loop-associated lncRNAs to form topologically associating domains (TADs) [115], yeast neither expresses CTCF orthologs [116] nor exhibits TADs [117]. Instead, chromatin looping in yeast is achieved through the recruitment of the chromatin remodelling complex, including the SMC (Structural Maintenance of Chromosomes) complex [118], the SWI/SNF (SWItch/Sucrose Non-Fermentable) complex [119], or the RSC (Remodeling the Structure of Chromatin) complex [120]. In fact, the RSC complex is involved in the case of gene looping mediated by the *CDC28* antisense lncRNA. This lncRNA is located within *CDC28,* which encodes for the cyclin-dependent kinase catalytic subunit regulating the cell cycle [121], and it is induced under osmotic stress [122]. Specifically, *CDC28* lncRNA plays a role in recruiting stress-activated protein kinase Hog1p to promote the expression of the *CDC28* sense gene via mediating gene looping [123]. Hog1p is induced in high osmotic stress to regulate a great number of downstream stress-responsive genes, including *CDC28* [124,125]*.* Hog1p can also trigger chromatin remodelling to promote polymerase progression by recruiting the chromatin remodelling complex RSC [126] and RNA polymerase II [123,127]. *CDC28* transcription is highly positively correlated with the abundance of Hog1p docked in the +1 nucleosome regions of *CDC28* [123]. The +1 nucleosome remodelling by the RSC complex, recruited by Hog1p, is required for the transcription initiation of *CDC28*. However, Hog1p is not directly recruited to the +1 nucleosome by the* CDC28* gene itself. Instead, its recruitment is initiated by the *CDC28* lncRNA transcription, which binds Hog1p, RSC complex and Ssu72p at the 3′ UTR of the *CDC28* gene on the chromatin. Next, an Ssu72-mediated gene-looping is triggered to facilitate the transferring of Hog1p from 3′ UTR of the *CDC28* gene to the +1 nucleosome [128]. In the absence of *CDC28* lncRNA, Hog1p aggregates on the 3′ UTR of the *CDC28* gene but fails to transfer onto the +1 nucleosome, which results in a low transcription of *CDC28*. In contrast with the TI, in the lncRNA-mediated gene looping, the lncRNA transcript is necessary for the repression of genes. It has been shown that the insertion of a terminator downstream of the *CDC28* lncRNA promoter blocks the expression of the *CDC28* lncRNA and consequently prevents the chromatin remodelling and the recruitment of Hog1p at the +1 nucleosome region [123].

Finally, a fourth lncRNA *cis*-regulation has been identified for some antisense lncRNAs that form DNA/RNA or RNA/RNA hybrid structures [129]. Such structure can (i) inhibit the access of regulatory elements, (ii) defect the DNA replication by blocking replication fork, or (iii) create double-strand breaks that trigger the accumulation of mutations and increase chromatin instability [130]. An example of lncRNA that forms DNA/RNA hybrids is TERRA, a sub-class of lncRNA initiated from the C-rich region of sub-telomeres by RNA polymerase II (Figure 3, Panel D)[45]. TERRA lncRNA sequence is enriched with the guanine nucleotide, which allows the formation of a stable hybridisation to the C-rich telomere via hydrogen bond [131]. As a result, TERRA can anneal with the telomeric duplex and form a stable three-stranded RNA/DNA telomeric hybrid configuration, which is also called R-loops [132,133]. The resulting change in chromatin structure reduces the telomere accessibility to the telomerase, which prevents the telomere elongation (Figure 3, Panel D). Therefore, the transcription of TERRA lncRNAs is negatively correlated with the telomere length [134]. When chromosomes exhibit long telomeres, both non-hybridised and hybridised TERRA are constrained to a low abundance by being degraded with the 5′-3′ single-stranded RNA exonuclease Rat1p and RNase H2 respectively, which are both recruited by the local telomeric proteins Rap1p [133,135]. The number of Rap1p bound to the telomere is proportional to the length of the telomere; therefore, the more the telomere is long, the more the exonucleases Rat1p and RNase H2 are recruited. When the chromosomes exhibit short telomeres, the degradation of TERRA is impaired since there are naturally fewer Rap1p able to bind to the telomere. Thus, fewer nucleases are recruited, which consequently increases the TERRA levels. Notably, TERRA R-loops in short-telomere chromosomes trigger the activation of the DNA damage response and promote homology-directed repair with the recruitment of the Rad51p recombinase [133], which enables the telomere re-elongation. This regulatory mechanism triggers the degradation and accumulation of TERRA in long-telomere and short-telomere chromosomes, respectively, which is essential to prevent early senescence in long-telomere cells. In addition, TERRA lncRNAs are also suspected to directly inhibit the telomerase by forming RNA/RNA hybrid structures with Tlc1p [45], which encodes for the RNA subunit of telomerase in yeast [136]. It has also been demonstrated that human telomeric RNAs are able to inhibit telomerase activity [137].

## Mechanisms of lncRNA regulatory function in *S. cerevisiae* in *trans*

While many lncRNAs in yeast primarily act in *cis*, few cases where lncRNAs act in *trans* have been reported in *S. cerevisiae*. The *trans* mechanism usually involves lncRNAs recruiting regulatory elements to a specific genomic region in order to control gene expression.

**Figure 4:**
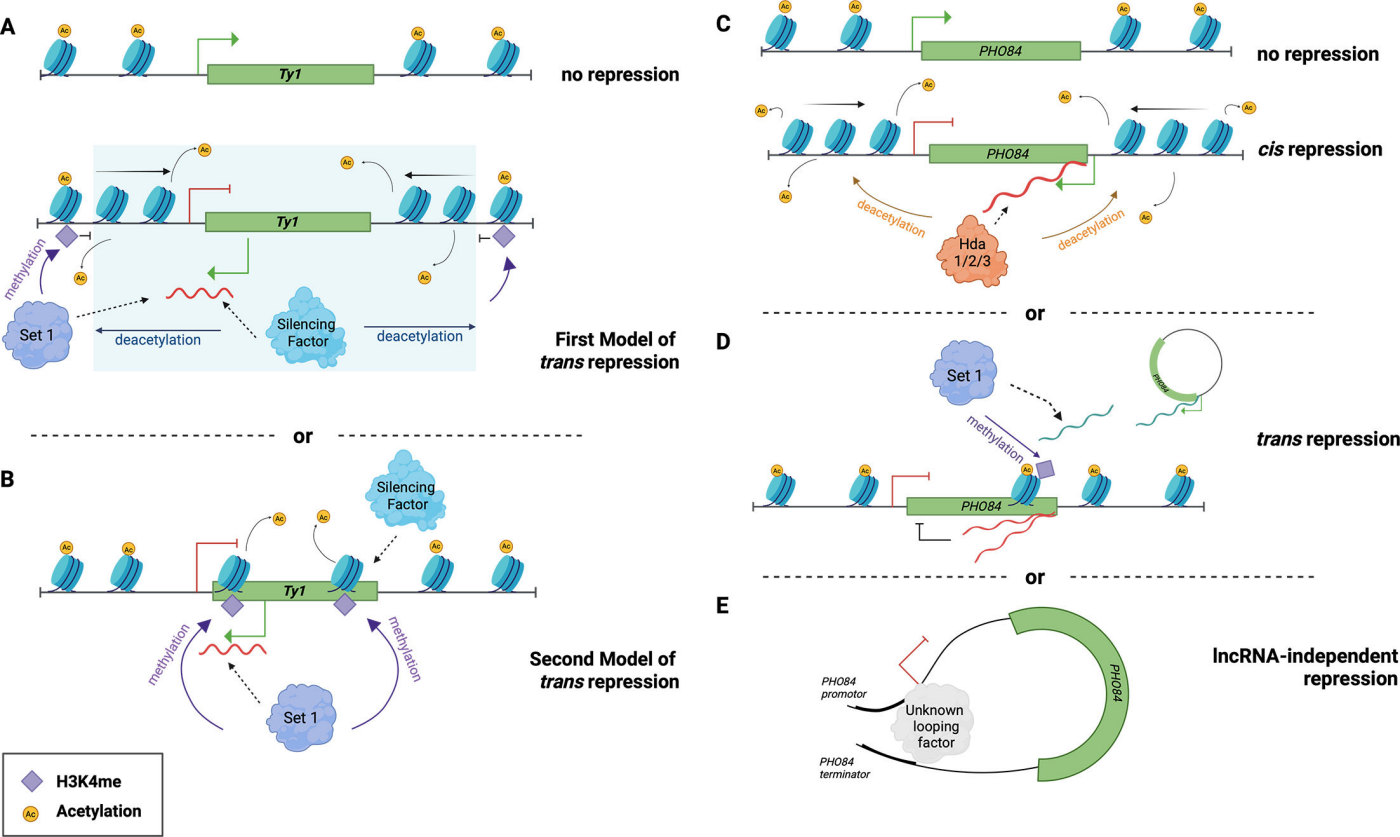
Mechanisms of trans-regulatory lncRNAs involved in *S. cerevisiae.* (**A**) The first proposed model of *Ty1 RTL*-mediated *Ty1* silencing. An unknown silencing factor is recruited by *Ty1 RTL* and inhibits *Ty1* transcription directly through histone deacetylation. Sen1p is also recruited by *Ty1 RTL* and methylates the border histone to limit the silencing factor within the *Ty1* locus (blue area). This methylation prevents the spread of histone deacetylation to the neighbouring genes. (**B**) The second proposed model of *Ty1 RTL*-mediated *Ty1* silencing. Sen1p is recruited directly b*y Ty1 RTL* and methylates the histone on the *Ty1* locus, which acts as a recognition site for the silencing factor for further histone deacetylation. (**C**) *PHO84* antisense RNA CUT281 represses *PHO84* in *cis* **.** On the one hand, CUT281 transcripts recruit the histone deacetylase Hda1/2/3, which removes the acetyl group from histones, which results in a tighter chromatin structure, thus inhibiting PHO84 transcription. (**D**) *PHO84* antisense RNA CUT281 represses *PHO84* in *trans*. On the other hand, Sen1p is recruited and conducts histone methylation on the *PHO84* ORF to promote the transcription of antisense RNAs, which silences *PHO84* in a *cis*-related mechanism. (**E**) Model of antisense RNA-independent *PHO84* repression. In addition, there is a new model of *PHO84* repression that proposes that the 3′ UTR and promoter regions of *PHO84* form a 3′UTR-dependent gene loop mediated by an unknown looping factor. The figure was created using BioRender (https://biorender.com/) *.*

For example, the *Ty1* antisense RNA, also known as *Ty1* CUT or *Ty1 RTL* RNA, acts in *trans* to repress the transcription and the mobility of the retrotransposon *Ty1* through a mechanism involving both histone deacetylation and H3K4 methylation [40,138]. In *S. cerevisiae*, *Ty1* is regulated by co-repression, similarly to siRNAs in plants, where the silencing of the gene is homology-dependent and induced by the high gene copy number of the gene itself [139,140]. The *Ty1 RTL* is transcribed from the 5′-end long terminal repeat region of the *Ty1* gene*.* The order of histone deacetylation and methylation on *Ty1* can be supported by two models. In the first model (Figure 4, Panel A), the *Ty1 RTL* targets an unknown silencing factor that inhibits the *Ty1* transcription through histone deacetylation, followed by a Set1/5-dependent histone H3K4 methylation to restrict the silencing factor on the *Ty1* locus. As a result, these methylations prevent the spread of the deacetylation along the neighbouring genes. In the second model (Figure 4, Panel B), the *Ty1 RTL* recruits the Set1/5-dependent histone H3K4 methylation, which directly triggers the *Ty1* transcriptional gene silencing. First, Set1p confers histone methylation on the *Ty1* locus, which is then recognised by the unknown silencing factor that represses the *Ty1* transcription through histone deacetylation. The *trans* activity of *Ty1* has been experimentally validated via its expression from a plasmid that resulted in the repression of the *Ty1* gene activity [40].

Another documented example of lncRNA acting in *trans* is SUT457, which plays a role in telomere overhang homeostasis [141]. Telomere homeostasis is reliant on the existence of the 3′-end ssDNA on telomeres (i.e. telomeric overhangs) [142]. Telomeric ssDNA accumulation is a cause of telomere degradation [143,144]. It was reported that SUT457 interacts with 12 telomere organisation genes and regulates the Exo1-dependent accumulation of telomeric ssDNA [141]. The deletion of SUT457 has been shown to promote cell senescence, and the relocation of SUT457 in other parts of the genome doesn’t affect the regulation of the telomeric ssDNA overhang length, which emphasises the *trans-*action of SUT457 [141]. However, the molecular mechanism of *trans*-regulation for this lncRNA remains unknown.

There is evidence that one lncRNA, CUT281, is able to perform regulatory functions in both *cis* and *trans.* CUT281 is the antisense transcript of *PHO84*, a gene encoding an inorganic phosphate transmembrane transporter. CUT281 has been shown to act in *cis* by recruiting the histone deacetylase complex Hda1/2/3 on *PHO84*, resulting in histone deacetylation. This causes the formation of a tight chromatin structure, which prevents the *PHO84* transcription (Figure 4, Panel C) [109]. In addition, CUT281 has also been reported to *trans*-regulate *PHO84* by dose-dependent co-repression as described for the *Ty1* silencing (Figure 4, Panel D) [145]*.* Contrarily to the *cis*-regulation, the repression mechanism in *trans* is independent of Hda1/2/3 but is instead mediated by Set1-dependent histone methylation. Specifically, Set1p conducts H3K4me2 and H3K4me3 in the middle of the *PHO84* ORF. However, instead of repressing the *PHO84* gene transcription directly, such histone methylation is suggested to promote the transcription of antisense RNAs, which silence *PHO84* in *cis*. This suggests that the *cis-* and *trans-*regulation of *PHO84* by CUT281 may be co-operative. It has been shown that the expression of *PHO84* from a plasmid results in the silencing of both exogenous and endogenous *PHO84* genes, supporting the fact that CUT281 can act in *trans* [145]*.* Nevertheless, recent research has questioned the previously described role of antisense RNA in repressing the *PHO84* gene. Transient-transcriptome sequencing in yeast revealed that *PHO84* gene repression is independent of *PHO84* antisense RNA, since a positive correlation exists between the sense and antisense transcripts on the *PHO84* locus [146]. It has been shown that the deletion of *PHO84* 3′ UTR prevents the silencing of *PHO84*, even though the antisense RNAs are present at the same levels as in the wildtype. This suggests that 3′ UTR, rather than *PHO84* antisense RNA, is key for gene repression. Another study suggested a model where *PHO84* silencing is driven by the process of 3′ UTR transcription and where *PHO84* antisense RNAs experience early transcription termination by NNS complex [82]. Disruption of NNS function increases the transcriptional output of *PHO84* antisense RNAs, leading to enhanced repression of the *PHO84* sense gene. However, no corresponding accumulation of antisense RNAs is observed at the locus while *PHO84* sense gene is repressed, as they are rapidly exported to the cytoplasm. This suggests that *PHO84* silencing is driven by the process of antisense transcription traversing the promoter region, rather than by the mere presence or buildup of antisense RNAs at the sense locus. A new model has been proposed that the 3′ UTR and the promoter region of *PHO84* form 3′ UTR-dependent gene loops, which triggers the *PHO84* repression (Figure 4, Panel E) [146].

In recent years, more lncRNAs regulating gene transcription in *trans* have been identified by knocking out lncRNAs systematically in the genome and complementing their expression using plasmids. These are SUT075 [147], SUT125, SUT126, SUT035 and SUT532 [148]. Expanding the catalogue of *trans*-regulatory lncRNAs is crucial in unravelling their diverse and functional roles in modulating transcriptional networks across the genome.

## Antisense lncRNAs: emerging key players in gene regulation

Antisense lncRNAs represent a pervasive class of transcripts with regulatory potential that are transcribed from the strand opposite to protein-coding genes [149]. Based on their TSSs, antisense lncRNAs can overlap with the 3′ UTRs, ORFs and promoter regions of the corresponding sense genes, or simultaneously overlapping multiple of these regions [102]. Antisense lncRNAs mainly consist of SUTs (~50%) [67], but also include other classes of ncRNAs such as CUTs [42,43], XUTs [44] and NUTs [52]. As mentioned in the previous sections, antisense lncRNAs play crucial roles in fine-tuning gene expression through multiple *cis-* (e.g. *GAL10* lncRNA) or *trans-* (e.g. *PHO84* lncRNA) mechanisms, often in a locus-specific and condition-dependent manner [150].

Growing evidence suggests that antisense lncRNAs represent a ubiquitous and functionally relevant class of gene regulator. First, genes that also have antisense transcripts exhibit higher expression variability and are more frequently switched off [67]. Thirty percent of the genes that are repressed during the exponential phase of growth possess antisense lncRNAs overlapping their promoters, suggesting that antisense-mediated repression is a widely employed mechanism under specific conditions [150]. In addition, antisense lncRNAs extending into promoter regions have been reported as a characteristic feature of SAGA-dependent (Spt-Ada-Gcn5-acetyltransferase-dependent) genes [151], a class of genes typically associated with ‘closed’ promoters and often transcriptionally inactive until induced by stress [152]. This regulatory effect is thought to be mediated by the recruitment of the histone regulatory complex HIR [151]. Moreover, a global anti-correlation has been observed between sense mRNA abundance and antisense transcription levels, supporting the repression regulatory role of antisense lncRNAs [60]. These observations suggest a widespread regulatory role of antisense transcription in modulating sense gene expression.

As discussed in the previous sections (i.e. ‘mechanisms of lncRNA regulatory function in *S. cerevisiae* in *cis*/*trans*’), antisense lncRNAs have been observed to employ histone methylation and/or deacetylation around the sense gene promoter region to repress its expression [109,110]. In fact, when antisense lncRNAs are transcribed by RNA polymerase II and extended into the 5′ sense promoter, co-transcriptional modifications, such as H3K36 methylation and histone deacetylation, occur on the traversed chromatin, thereby repressing transcription initiation at the overlapping sense promoters [153,154]. Therefore, it is hypothesised that the relationship between sense-antisense is antagonistic, and a global negative correlation is expected between sense and antisense transcription. However, this view has been challenged by the observation of a weak relationship between the levels of nascent sense and antisense transcription in the yeast population, suggesting that the transcription of antisense lncRNAs is regulated independently of the sense transcription [155]. A later study revealed that sense and antisense transcription indeed exhibit distinct chromatin environment signature, including different nucleosome occupancy, histone modification and histone turnover rates in a single cell [156]. Specifically, sense transcription typically correlates with open chromatin features such as high levels of H3K36me3, H3K79me3 and H2B ubiquitination, whereas antisense transcription is linked to closed promoter configurations, increased nucleosome occupancy, higher histone acetylation and elevated histone turnover. These contrasting chromatin states suggest that the sense and antisense transcription are regulated independently and tend to occur in separate cells in the population, thereby masking the inverse relationship at the population level, supporting the antagonistic relationship between sense-antisense transcription [156].

The regulatory mechanisms of antisense lncRNAs cover TI, recruit regulatory elements and gene looping (see section ‘mechanisms of lncRNA regulatory function in *S. cerevisiae* in *cis*’). It is also reported that the transcription of antisense lncRNAs impacts the dynamics of sense transcription, i.e. production, processing and degradation rate, through the modulation of the chromatin environment [157]. Chromatin environment signatures of antisense lncRNAs alter the sensitivity of the sense gene to antisense lncRNAs unrelated chromatin modification. For example, it is the case with Set3p, where the highly expressed antisense lncRNA reduces the sense gene’s sensitivity to Set3p deacetylation. This highlights the complex regulatory relationship between antisense lncRNAs and sense gene expression.

Several studies have reported that many antisense lncRNAs in yeast can form double-stranded RNA by base-pairing with overlapping mRNAs [53,158,159]. Therefore, a new perspective in yeasts proposes that antisense lncRNAs can promote the expression of the corresponding sense gene. The formation of double-stranded RNAs possesses higher stability and promotes mRNA export into cytoplasm in *S. cerevisiae* [160]. A similar phenomenon has been observed in fission yeast, where highly expressed genes appear to be particularly sensitive to antisense RNA-mediated regulation [58]. Such a perspective proposed a new *trans-*acting mechanism of lncRNAs to modulate gene expression beyond local transcriptional regulation, expanding our understanding of the functional versatility of lncRNAs in yeast and potentially other eukaryotes.

## Functional profiling of ncRNAs in yeast

In yeast, there is still a large number of ncRNAs for which the function remains underexplored. Functional profiling of yeast ncRNAs aims to systematically characterise these transcripts by identifying how their expression, interactions and disruption influence the fitness, stress responses and broader regulatory networks within the cell.

Functional profiling by phenotypic screening of yeast knockout or overexpression strains has proven to be a robust approach for understanding the function of ncRNAs, since yeast is amenable to genetic manipulation [161,162]. Large-scale fitness screenings from solid medium plates are facilitated thanks to automated high-throughput robots, such as the Singer Instrument RoToR HAD, and by advanced image-processing pipelines, such as Balony [163]. Using this approach, a collection of 1502 *S. cerevisiae* ncRNA deletion strains has been generated [147,164]. This barcoded ncRNA gene deletion library includes 743 haploids (373 *MAT* **a** and 370 *MATα*) and 759 diploids (331 homozygotes and 428 heterozygotes) strains. Fitness profiling was performed via genome-wide competition experiments across eight stress conditions, which revealed environment-specific effects of ncRNA deletions, as well as identified new essential ncRNAs in rich medium YPD at 30°C (SUT075, SUT367, SUT527 and SUT259/691). Thanks to this resource, the lncRNA SUT527 has been functionally characterised. SUT527 overlaps with the 3′ UTR of the essential gene *SEC4* that encodes GTPase, and its knockout impacts *SEC4* mRNA processing and localisation [147]. It has been shown that SUT527 forms a double-stranded RNA with *SEC4* transcript, which affects not only the SEC4 mRNA stability but also the 3′ end formation and localisation. In another study from Balarezo-Cisneros [148], a phenotypic screening of the *S. cerevisiae* ncRNA deletion library was carried out in 23 different conditions, which revealed four ncRNA deletions (SUT125, SUT126, SUT035 and SUT532) that caused fitness defects across the majority of conditions tested [148]. Complementation experiments of these four ncRNA knockouts using plasmids revealed that their regulatory mechanism acts in *trans*. Further transcriptome analysis of these four mutant strains showed their role in regulating transcription factors [148]. In the fission yeast *S. pombe,* fitness screening on 141 lincRNA deletion mutants revealed that *SPNCRNA.989* and *SPNCRNA.1343* play roles in regulating phosphate homeostasis by repressing neighbouring gene *ATD1* (encoding for aldehyde dehydrogenase) and *TGP1* (encoding for transmembrane transporter), respectively. Phenotyping of lincRNA overexpression mutants uncovered three new lncRNAs, *SPNCRNA.1154, SPNCRNA.1530* and *SPNCRNA.335*, which play roles in the meiotic differentiation [165]. Moreover, the integration of structure-based screening with *in vivo* functional characterisation of *S. pombe* ncRNA knockouts unveiled the role of the lncRNA nc1669 in the repression of untimely sexual differentiation [166].

Besides growth fitness screenings, ncRNA functional profiling can also be achieved by examining the cell morphology of ncRNA knockout strains. For example*,* high-throughput microscopy and flow cytometry assays on *S. pombe* lincRNA deletant strains helped uncover the roles of 41 lincRNAs in cell-size regulation and/or cell cycle [165]. Specifically, two lincRNA mutants, *SPNCRNA.819Δ and SPNCRNA.989Δ*, have been shown to have a significantly shorter binucleated cell length compared with the wildtype cells, while four lincRNAs, *SPNCRNA.323, SPNCRNA.943, SPNCRNA.412 and SPNCRNA.236,* exhibit longer binucleated cell length, suggesting their involvement in the co-ordination of cell growth and division [165]. In addition, in another study characterising the regulatory effects of 162 antisense SUTs, unidirectional transcriptional repression has been applied on antisense SUTs [167]. Out of the 162 tested antisense SUTs, 41 were found to alter the protein abundance of their corresponding sense genes [167]. This set included the previously characterised *IME4* antisense lncRNA (known for its negative *cis*-regulatory function) and the antisense lncRNA of *AMS1* (encoding for vacuolar α-mannosidase), which is a rare example of positive regulation between an antisense lncRNA and its sense gene. It has been shown that the regulatory effects of antisense lncRNAs correlated with TSS overlap between sense and antisense transcripts and were facilitated by H3K4 methylation.

Additionally, ncRNA functions can be explored through synthetic genetic array (SGA) analysis, which examines genetic interactions (GIs) between target ncRNAs and other ncRNAs or protein-coding genes [168]. This approach allows researchers to infer cellular roles by examining the GI profiles of specific ncRNAs. For example, an SGA analysis was carried out in *S. cerevisiae* on six intergenic SUT queries with the array of 4309 non-essential gene deletion mutants [141]. The negative epistasis network of these six SUTs with different genes revealed a diverse gene ontology enrichment that could pinpoint their roles in the cell, such as (i) telomere organisation for SUT457, (ii) vesicle-associated functions for SUT042, (iii) mitochondrial functions and chromatin organisation among others for SUT014, (iv) organelle fission among others for SUT469, and (v) histone modification among others for SUT451. For SUT123, the negative epistasis network did not have a significant enrichment in any gene ontology categories. Notably, the neighbouring genes of SUT457 and SUT042 possess different functions compared with the enrichment of the genes interacting with them, suggesting that they might act in *trans*. In fact, SUT457 was confirmed as a *trans*-actor crucial for telomeric overhang homeostasis via an Exo1-dependent pathway [141].

## Transcriptional profiling of ncRNAs in yeast

Transcriptional profiling is an alternative way to profile ncRNA functions, providing a global overview of genomic dynamics. Transcriptional profiling allows the capture of changes in RNA expression that could affect the cellular function without necessarily affecting the overall fitness. Genome-wide expression profiles between different strains in one or multiple conditions can reveal the global transcriptomic fingerprint of a candidate ncRNA and assist the characterisation of ncRNA in terms of cellular function, GI or regulatory networks [95,148,169].

Most of the transcriptional profiling aims at identifying ncRNA profile fingerprints in different cell types or conditions. For example, a transcriptome study on quiescent and G_1_ yeast cells has revealed ncRNA transcriptional profiles between the two-cell stages [170]. It has been shown that there are more ncRNA transcripts present in the quiescent transcriptome than in the G_1_ transcriptome. Additionally, 241 ncRNAs were exclusively expressed in quiescent cells, while 42 ncRNAs were found to be expressed exclusively in G_1_ cells.

Moreover, the difference in ncRNA transcriptional profiles was also studied between differentiated cell subpopulations of either yeast colonies or biofilms [171]. Expression of ncRNA classes exhibits cell-type-specific patterns. Specifically, SUTs constitute the majority of differentially expressed (DE) lncRNA in spatial and temporal comparison, MUTs are primarily DE in biofilm colonies versus smooth colonies (20% to 6.5%), and CUTs exhibited up-regulation in the yeast colonies over time. In addition, regarding the spatial arrangement of lncRNAs concerning neighbouring gene pairs, the antisense lncRNAs that overlap with genes or originate from the same promoter regions emerge as the most common category showing co-regulation (agonistically differential expression) or anti-regulation (antagonistically differential expression) with paired genes, highlighting the significance of antisense lncRNA in positive or negative regulation of neighbouring genes during biofilm formation. The study not only suggests the diverse function of ncRNA in different cell subpopulations but also provides insight into the specific cellular functional categorisation of different lncRNA classes.

Besides *S. cerevisiae,* transcriptional profiling is also performed in other yeast species by comparing gene expression patterns across different strain sources or environmental conditions. In the *Candida* genus, expression profile analysis on lncRNAs of four *Candida* species (*C. albicans*, *C. tropicalis*, *C. parapsilosis* and *C. glabrata*) has discovered a group of infection-specific lncRNAs with the potential role of *Candida* lncRNAs in virulence [95]. In this study, the transcriptional profile uncovered a subset of lncRNAs, ca. 191–270 in number depending on the species, which display infection-specific expression patterns and indicate their potential role in the infection process. These infection-specific lncRNAs also participate in some co-expression modules that are enriched in terms linked to pathogenicity. Additionally, the co-expression analysis with the lncRNA subset and protein-coding genes identified enrichments in biological functions linked to fungal pathogenicity, supporting the hypothesis that these lncRNAs may play roles in fungal infection and pathogenesis.

Researchers also applied expression profiling on ncRNA deletion mutants to investigate their impact on the transcriptome and networks. In the study of Balarezo-Cisneros [148], it was reported that lncRNAs in *S. cerevisiae* can exert context-dependent phenotypes and influence the protein regulatory network in *trans* [148]*.* Among the 18 tested lncRNA deletants, more than one-third had extensive transcriptional alterations, affecting hundreds of coding and non-coding RNAs. In addition, the transcriptional profile of heterozygous deletions of the two essential ncRNAs, SUT075 and snR30, highlighted their roles in multiple cellular processes like ribosome biogenesis, rRNA processing, DNA replication and the cell cycle. These findings underscore the complexity of lncRNA genome-wide interactions within cellular systems, suggesting that these molecules can modulate gene expression not only through direct interactions with local chromatin or transcription factors but also by affecting the broader protein landscape of the cell.

Besides specific studies aiming to identify ncRNA transcriptional profiles, it is likely that a bulk of useful ncRNA transcriptional data is buried, and hence overlooked, in other gene-orientated transcriptomics studies. The lack of attention on ncRNAs in the past has limited our availability to systematically investigate their dynamic expression profiles. It will be beneficial to create a comprehensive, systematic and comparative transcriptomics database for yeast ncRNAs to enable more extensive comparative genomics studies and to facilitate future research on ncRNAs and their impact on various biological processes.

## ncRNAs: more than just noise in both transcriptomics and proteomics

ncRNAs were traditionally considered unlikely to be translated, hence their name ‘non-coding’. This classification was based on computational modelling, which took into account the absence of homology with known proteins, the small-sized open reading frames (ORFs<300 nt), and the low sequence conservation [172]. However, there is a growing amount of evidence reporting that some ncRNAs can be translated into small peptides less than 100 aa in *S. cerevisiae, Drosophila*, zebrafish, mice and humans [50,173–176] thanks to techniques such as ribosome profiling (Ribo-seq) and mass spectrometry. These ncRNAs were re-categorised into protein‐coding small open reading frames (smORFs) [177]. smORFs can originate from coding transcripts (5′ UTR, coding sequence, or 3′ UTR) or ncRNAs [178]. In vertebrates, the microproteins translated from smORFs can play roles in multiple cellular regulatory pathways, including membrane pump inhibition, muscle signalling [179], protein complex assembly [176], and cardiac functioning [180]. In addition, microproteins are also reported in microbes, such as *Escherichia coli, Bacillus subtilis* and *S. cerevisiae*, that function in antibacterial production [181], membrane regulation [182] and respiration [183], among others.

To date, identifying smORFs and microproteins remains challenging. Ribo-seq analyses often detect a large number of putative smORFs; however, many exhibit low expression levels, limited conservation and poor reproducibility [184,185]. Furthermore, ribo-seq alone does not provide direct evidence for the actual existence of translated proteins. ncRNAs may transiently associate with ribosomes but do not necessarily translate into stable proteins unless a triplet periodicity pattern is observed, which is unlikely for short ORFs. In proteomics, detecting microproteins using MS/MS spectra is difficult due to their rapid degradation [185], small size and low translation levels [186].

In yeast, lncRNAs possessing protein-coding potential have been increasingly reported. For example, a subset of uRNAs was found to possess protein-coding capacity and therefore be smORFs [50]. Many of these smORFs are conserved across fungal species, suggesting that the resulting polypeptides may play important biological roles in the cell. In addition, XUTs have been recently reported to be pervasively translated as they are bounded by ribosomes [86]. In contrast with canonical ORFs, which exhibit a strong bias toward the coding frame and display characteristic triplet periodicity, ORFs originating from non-coding regions show a more even distribution of ribosome footprint reads across all three frames [187]. Due to this atypical ribosome occupancy pattern, these ORFs are also described as non-canonical translated RNAs or non-canonical ORFs (nORFs).

The observation of nORFs in yeast raises an important question about their evolutionary significance and the mechanisms that preserve them. Most nORFs are evolutionarily young (or ‘transient’) and lack evidence of purifying selection [185], suggesting that they are not likely to be maintained in the genome. Moreover, many nORFs are cryptic [86], making them appear to be byproducts of low-fidelity protein synthesis. Consequently, similarly to lncRNAs being referred to as transcriptional noise, nORFs have been referred to as translational noise. However, it has been reported that nORFs can generate stable protein products and are involved in cellular networks, affecting cellular phenotypes [185,188], suggesting these ‘transient translatome’ is functional [189]. In addition, it has been widely suggested that nORFs play an important role in *de novo* gene birth [185,190,191]. It has been revealed that the majority of intergenic ORFs in *S. cerevisiae* possess the fundamental structural components of proteins, suggesting the structural potential of nORFs to form *de novo* proteins [190]. Strikingly, the potential of *de novo* gene birth from nORFs is facilitated by NMD. The potentially deleterious effects of peptides translated from nORFs are mitigated by the NMD pathway, which efficiently targets and degrades these transcripts [87], thus encouraging sampling of new ORFs [50]. In addition, nORFs can serve as a pool for generating proto-genes, which facilitates *de novo* gene birth and provides adaptive potential under stress conditions. Non-genic ORFs that are found exclusively in the *Saccharomyces* genus are believed to have emerged recently. They exhibit strong divergent patterns of translation between starvation and nutrient-rich conditions [191]. In contrast, the more conserved non-genic ORFs among fungi species lack such condition-specific translation patterns. This suggests that newly emerged ORFs may contribute to adaptive functions in response to environmental stress [191]. Hence, a model has been proposed in which non-genic ORFs, proto-genes and functional genes exist on a continuum without strict annotation boundaries [191].

In sum, the boundary between translated lncRNAs and mRNAs is now challenged. The discovery of nORFs and evidence of their functionality introduces a novel perspective on lncRNA function, highlighting their potential role as a source of genetic innovation through the production of new peptides.

## Conclusions and outlook

Recent advances in transcriptomic technology have led to the discovery of diverse classes of ncRNAs in yeast and illuminated their cellular fate from transcription to degradation. ncRNAs play essential roles in gene regulation, both in *cis* and *trans*. Functional and transcriptional profiling has revealed a wide range of cellular functions, GIs and regulatory networks governed by ncRNAs, underscoring their importance in cellular dynamics. Moreover, emerging research has challenged the traditional definition of ncRNAs, showing that some may have the potential to be translated into proteins.

However, there is still a great gap in understanding the comprehensive functional roles and mechanisms of action for ncRNAs, especially the class-specific functions or mechanisms (e.g. antisense lncRNAs). Additionally, the global interaction networks of ncRNAs with other cellular components (such as protein-coding genes, other ncRNAs, transcription factors and more) remain insufficiently explored. Future studies could focus on mapping both physical and GI networks of ncRNAs, particularly those involving chromatin modifications and other epigenetic mechanisms that are currently overlooked. Finally, the evolutionary origins and conservation of yeast across different species would be an interesting target to characterise ncRNAs. Understanding their evolutionary origins and conservation would help to uncover whether certain ncRNAs have unique, species-specific roles or represent conserved regulatory mechanisms since ancient ages. Future studies will be answering some of these complex questions and will advance our understanding of ncRNA roles across species.

## Abbreviations

CD-CUTs: cytoplasmically degraded cryptic unstable transcripts
CDK: cyclin-dependent kinase
CID: CTD interaction domain
CPF-CF: cleavage and polyadenylation/cleavage factor
CTD: C-terminal domain
CUTs: cryptic unstable transcripts
DE: differentially expressed
GIs: genetic interactions
MAT: mating type
MUTs: Meiotic Unannotated Transcripts
NFRs: nucleosome-free areas
NMD: nonsense-mediated decay
NMD: nonsense-mediated decay
NNS: Nrd1-Nab3-Sen1
ORF: open reading frame
ORF-NFRs: open reading frames NFRs
RUFs: transcripts of ncRNA of unknown function
RUTs: Reb1p-dependent unstable transcripts
SCR1: small cytoplasmic RNA
SGA: synthetic genetic array
SUTs: stable unannotated transcripts
TADs: topologically associating domains
TERRAs: telomeric repeat associated cryptic ncRNAs
TI: transcriptional interference
TMLs: telomerase-mutant lncRNAs
TSS: transcription start site
UTR: untranslated region
XUTs: Xrn1-sensitive unstable transcripts
YPD: yeast extract peptone dextrose
aa: amino acids
lincRNAs: long intergenic non-coding RNAs
lncRNA: long non-coding RNA
nORF: non-canonical ORFs
ncRNA: non-coding RNAs
smORFs: small open reading frames
snRNAs: small nuclear RNAs
snoRNAs: small nucleolar RNAs
ssDNA: single-stranded DNA
uRNAs: unannotated RNAs
